# Characterization and Fine Mapping of a Yellow-Virescent Gene Regulating Chlorophyll Biosynthesis and Early Stage Chloroplast Development in *Brassica napus*

**DOI:** 10.1534/g3.120.401460

**Published:** 2020-07-09

**Authors:** Chuanji Zhao, Lijiang Liu, Luqman Bin Safdar, Meili Xie, Xiaohui Cheng, Yueying Liu, Yang Xiang, Chaobo Tong, Jinxing Tu, Junyan Huang, Shengyi Liu

**Affiliations:** * Oil Crops Research Institute of the Chinese Academy of Agricultural Sciences/The Key Laboratory of Biology and Genetic Improvement of Oil Crops, The Ministry of Agriculture and Rural Affairs, Wuhan 430062, China; † National Key Laboratory of Crop Genetic Improvement, National Center of Rapeseed Improvement, Huazhong Agricultural University, Wuhan 430070, China; ‡ Guizhou Rapeseed Institute, Guizhou Academy of Agricultural Sciences, Guiyang 550008, P. R. China

**Keywords:** *Brassica napus*, leaf color, chlorophyll biosynthesis, chloroplast development, BSA-seq, gene cloning, RNA-seq

## Abstract

Chlorophyll biosynthesis and chloroplast development are crucial to photosynthesis and plant growth, but their regulatory mechanism remains elusive in many crop species. We isolated a *Brassica napus* yellow-virescent leaf (*yvl*) mutant, which exhibited yellow-younger-leaf and virescent-older-leaf with decreased chlorophyll accumulation and delayed chloroplast development. We mapped *yvl* locus to a 70-kb interval between molecular markers yvl-O10 and InDel-O6 on chromosome A03 in BC_2_F_2_ population using whole genome re-sequencing and bulked segregant analysis. The mutant had a ‘C’ to ‘T’ substitution in the coding sequence of *BnaA03.CHLH*, which encodes putative H subunit of Mg-protoporphyrin IX chelatase (CHLH). The mutation resulted in an imperfect protein structure and reduced activity of CHLH. It also hampered the plastid encoded RNA polymerase which transcribes regulatory genes of photosystem II and I. Consequently, the chlorophyll a/b and carotenoid contents were reduced and the chloroplast ultrastructure was degraded in *yvl* mutant. These results explain that a single nucleotide mutation in *BnaA03.CHLH* impairs PEP activity to disrupt chloroplast development and chlorophyll biosynthesis in *B. napus*.

Chlorophyll biosynthesis is an important component of tetrapyrroles’ synthetic metabolism (Czarnecki and Grimm 2012), and disruption in this pathway can induce changes in pigments and leaf color, such as chlorotic leaves (Wu *et al.* 2007). Chlorotic leaves disrupt the photosynthetic capacity of plants resulting in damages to plant metabolism and ultimately yield products, and hence, several studies have evaluated the genetic mechanism underlying chlorotic leaves in crop plants (Yang *et al.* 2016; Ge *et al.* 2017). In a previous study, Gibson and colleagues reported that the conversion of protoporphyrin IX into Mg-protoporphyrin IX was possible when three genes *BchH*, *BchD* and *BchI* were expressed together, and mutations in these plant genes caused disruptions in chlorophyll biosynthesis (Gibson *et al.* 1995). Afterward, the various subunits of chlorophyll biosynthesis genes were studied in several crop plants, such as *OsCHLH*, *OsCHLD* in rice and *GhCHLI* in cotton (Jung *et al.* 2003; Ruan *et al.* 2017; Zhu *et al.* 2017a). Apart from its role in chlorophyll biosynthesis pathway, Mg-protoporphyrin IX chelatase is also involved in retrograde signaling. Retrograde signaling is a complex network that regulates chloroplast development during the early leaf stage and transfers information about the status of chloroplast to the nucleus to regulate gene expression (Pogson and Albrecht 2011; Hernández-Verdeja and Strand 2018). Earlier, Mg-protoporphyrin IX chelatase was suggested to be a plastid signal in retrograde signaling pathway that regulated the expression of photosynthesis-related nuclear genes (Mochizuki *et al.* 2001). A later study reported that Mg-protoporphyrin IX affected chlorophyll synthesis and diffusion, or shuttled chloroplast envelope to carry plastid signals into cell solutes (Barajas-Lopez Jde *et al.* 2013). *CHLH* gene coding the H subunit of Mg-chelatase is a key catalytic subunit in the production of Mg-protoporphyrin IX, and, therefore, an important component of chlorophyll biosynthesis and retrograde signaling (Moulin *et al.* 2008; Mochizuki *et al.* 2008).

Nuclear and chloroplast genes work in a coordinated manner to regulate chloroplast development, which is involved in the chloroplast and nuclear gene transcription, protein translation, processing modification, protein folding and degradation, thylakoid formation, and pigment synthesis (Beck 2001; Yu *et al.* 2013; Li *et al.* 2015). A cytoskeletal GTPase (*FtsZ*) is a component of plastid division machinery and is abundantly expressed in the early stage of chloroplast division (Vitha *et al.* 2001; TerBush *et al.* 2013). The transcription of plastid genes is primarily responsible for two RNA polymerases, the nuclear encoded RNA polymerase (NEP) and the plastid encoded RNA polymerase (PEP) (Shiina *et al.* 2005; Zubo *et al.* 2011). The PEP complex is composed of four NEP-encoded core subunits (*i.e.*, ropA, ropB, ropC1 and ropC2) and plays an important role in chloroplast maturity by producing many transcripts of photosynthesis genes (Borner *et al.* 2015).

*Brassica napus* (rapeseed) is a widely cultivated oil crop, mainly for its oil-rich seeds as it is the third largest vegetable oil source for humans. Although the chlorophyll biosynthesis genes have been well studied in other crops, such as *A. thaliana* and *O. sativa*, limited information is available for the regulatory mechanism of photosynthetic genes and yellowish leaf phenotype in rapeseed. A few leaf color mutants are reported (Wang *et al.* 2016b; Zhao *et al.* 2013) and only one gene, *BnaC07.HO1*, which encodes a heme oxygenase, has been cloned in *B. napus* (Zhu *et al.* 2017b). Apart from the involvement of *YVL* genes in photosynthetic machinery, *yvl* phenotype also plays a vital role as an indicator in cross breeding. During the crop production of hybrid rapeseed, the purity of male sterile line had decreased due to the influence of environmental factors or the mixing of maintainer lines in sterile lines, which further affected the purity of hybrid seeds. If the recessive leaf color marker traits, which are not only different from the normal green leaves but also well-developed in seedling stage and have little effect on yield, are introduced into sterile lines, false hybrids can be detected through leaf color at seedling stage (Zhao *et al.* 2010). However, most leaf color mutants lead to a functional disruption in chloroplasts and subsequently abnormal growth, sometimes even lethal effects on plants, which pose serious challenges to crop yield (Yang *et al.* 2016; Ge *et al.* 2017; Hsieh *et al.* 2017).

In this study, we isolated a mutant *yvl* with ethyl methanesulfonate (EMS) mutagenesis and successfully cloned the causal gene *BnaA03.yvl* with BSA-seq method. *BnaA03.yvl* caused a yellow-virescent leaf phenotype with chlorophyll deficient and disruptions in chloroplast structure. Also, mutations in *BnaA03.yvl* caused variation in transcript abundance levels of photosynthesis pathway genes. The results of present study explain the regulatory framework of chlorophyll biosynthesis pathway with *BnaA03.yvl* being a major contributor to the yellow-virescent leaf phenotype. Furthermore, the *yvl* mutant can also be used as a potential breeding marker in *B. napus*.

## Materials and Methods

### Plant materials and growth conditions

A yellow-virescent leaf (*yvl*) mutant of *Brassica napus* was isolated from ZS9 by treating with 0.5% EMS for 16 h. Two F_2_ populations, which had 927 and 346 individuals respectively, were generated from reciprocal crossing of *yvl* with ZS9. The F_1_ hybrid, *yvl*×ZS9, was three times backcrossed to *yvl*. One BC_1_F_1_ population of 159 lines and the two F_2_ populations were used for genetic analysis and BSA-seq. About 2200 progeny from self-crossing of BC_2_F_1_ were used for fine mapping. Twenty BC_3_F_1_ plants and a diversity panel of 629 oilseed accessions (Table S1), including ZS9 and *yvl*, were used to validate the substitution site in *BnaA03.YVL*. The 629 rapeseed accessions were worldwide collected and most of them came from China. All the plants were grown in fields located in the Hubei Province in central China.

### Chlorophyll determination

For pigment extraction, eight-leaf staged *yvl* mutant and ZS9 were sampled. The second and fifth leaves from the top were used as the younger and older leaves comprising about 30 mg of fresh weight. The leaves were then soaked in an 80% (v/v) acetone and ethanol mixture solution at 25° in dark for 48 h. Chlorophyll a (Chl *a*), chlorophyll b (Chl *b*) and carotenoid (Car) were measured with UV-spectrophotometer (ANALYTIKJENA), according to the method outlined by Arnon (Arnon 1949).

### Transmission electron microscopy analysis

The collected younger and older leaves were fixed in a phosphate buffer containing 2.5% (w/v) glutaraldehyde overnight at 4°, and then further fixed in 1% OsO_4_ at 4° for 3 h. Afterward, samples were successively distilled three times with saline phosphate buffer for 60 min, dehydrated with a series of gradient alcohol, treated with acetone and embedded in epoxy resins and polymerized at 60°. The samples were repaired and resembled at the appropriate size and shaped to slice about 60-100 nm with a microtome. The samples were stained with uranyl acetate and examined using Tecnai G^2^ 20 TWIN transmission electron microscope (FEI, U.S.A.).

### Whole genome re-sequencing and BSA-seq analysis

High-quality genomic DNA (gDNA) was isolated from fresh leaves by using a Hi-DNA secure Plant Kit (TIANGEN) and quantified to equal concentrations. For the rapid mapping and delimitation of the *yvl* locus, two parental lines and two extreme pools (*i.e.*, G-pool and Y-pool, each consisting of 30 individuals) were selected for the ZS9-type and *yvl*-type leaf colors from the F_2_ population of *yvl*×ZS9. Samples were prepared and subjected to WGS using Illumina HiSeq2500 platform and 125 bp paired-end reads were generated with an insert size of around 350 bp. Burrows-Wheeler Aligner (BWA) was used to align the clean reads of the two parents and two pools against the reference genome of *B. napus* (Li and Durbin 2009; Chalhoub *et al.* 2014). Alignment files were converted to Binary Alignment/Map (BAM) files using the Sequence Alignment/Map (SAM) tools software (Li *et al.* 2009). For all samples, SNP calling was performed by using the Unified Genotype function of the Genome Analysis Toolkit (GATK) software (McKenna *et al.* 2010).

To identify candidate genomic region responsible for *yvl*, we compared the ‘SNP-index’ between the G- and Y-pools. The SNP-index is estimated as a proportion of reads aligned to a position with a variant nucleotide different from the reference sequence. These positions were filtered out; SNP indexes in both pools were less than 0.3 and SNP sequencing depths were less than 7. The ΔSNP-index was calculated by subtracting the SNP-index of the G-pool from the Y-pool. A sliding window approach with 1 Mb window size and 10 kb step size as the default settings was used to measure the average distribution of all SNP-index of SNPs mapped across 19 chromosomes of *B. napus* in the given genomic interval. The distribution pattern of SNPs and InDels between ZS9 and *yvl*, as well as the SNP-index graphs for the G- and Y-pools, and corresponding ΔSNP-index graphs were plotted and showed by circular pictures.

### Fine mapping of the yvl locus

According to the WGS data, homozygous SNPs and InDels as polymorphic molecular markers were first identified by the alignment of reads from the parental lines to the reference genome using SAM tools. In order to confirm whether SNPs were homozygous and possibly polymorphic between the two parents, the primers (Table S2) were designed to amplify approximately 400-2000 bp nucleic acid sequences containing variations of SNPs and sequence the PCR products by the Sanger method to confirm positive polymorphisms (Wang *et al.* 2017). InDel primers (Table S2) were designed based on the flanking sequences of identified InDel positions. All of the primers were designed by using Primer 3.0 (version 0.4.0) online software (http://bioinfo.ut.ee/primer3-0.4.0/).

To obtain the *yvl* locus, 73 F_2_ and 544 BC_2_F_2_ individuals exhibiting the mutant phenotype derived from crossing *yvl* and ZS9 were constructed as mapping populations. The total genomic DNA was isolated from fresh young leaves of rapeseed seedlings using the cetyltriethylammnonium bromide (CTAB) mini-prep method (Murray and Thompson 1980).

Amplified fragments of InDel markers were subjected to electrophoresis on high resolution agarose gel and SNPs genotyping of mapping population were visualized with either the Sanger method sequence or Kompetitive Allele Specific PCR (KASP), a high throughput SNP detection technology (Semagn *et al.* 2013). The Laboratory of the Government Chemist (LGC) Group was employed to design the KASP primers, which matched the SNP alleles containing two different forward primers labeled by FAM and HEX with fluorophores and one same reverse primer (Table S2).

### Gene cloning and multi-sequence alignment

Genomic DNA of 14 open reading frames (ORFs) in the candidate region were amplified in ZS9 and *yvl* using gene-specific primers (Table S2) by using a PrimeSTAR GXL Kit (Takara, Japan) and cloned into the PBI121 Vector using ClonExpress II One Step Cloning Kit C112-01 (Vazyme). The recombinant plasmids were transformed into DH5α competent cells for sequencing. Multiple sequence alignment was performed with Clustal Omega online software (http://www.ebi.ac.uk/Tools/msa/clustalo/) and visualized by Sequence Manipulation Suite: Color Align Conservation (http://www.bioinformatics.org/sms2/color_align_cons.html).

### Phylogenetic and synteny analysis

The amino acid sequences of the CHLH homological proteins were retrieved through the *Brassica* Database (http://brassicadb.org/brad/index.php) and the plant genomics resource Phytozome v12.1 (https://phytozome.jgi.doe.gov/pz/portal.html) by searching with the total amino acid sequences of CHLH. The multiple sequence alignments were conducted by ClustalX 1.83 (Thompson *et al.* 1997). Phylogenetic trees were constructed using MEGA 5.1 software based on the neighbor-joining (NJ) method with 1000 bootstrap trials and 111 random seed (Tamura *et al.* 2011).

Extract the extended upstream and downstream 30 kb of the orthologous regions covering the *CHLH* genes from the reference genome in *Arabidopsis*, *B. napus*, *B. rapa* and *B. oleracea*. Perform blast alignments between each species to obtain a synteny region. The visualization of synteny region was exported from Circos software.

### RNA extraction, RNA analysis and qRT-PCR

Total RNA was extracted from the freshly prepared younger and older leaves at the eight-leaf stage using the RNA prep pure plant kit (TIANGEN). The RNA samples were diluted to 10 ng μl^-1^ and analyzed by Agilent 2100 (Agilent, U.S.A.). The RNA 6000 Nano Total RNA analysis Kit (Agilent, U.S.A.) was used for analysis.

Each RNA sample (2 μg) for quantitative RT-PCR (qRT-PCR) was reverse transcribed using PrimeScript RT reagent Kit with gDNA Eraser (Takara, Japan) following the manufacturer’s instructions. The *BnaActin* gene (GenBank: AF111812.1) was used as an internal control. The qRT-PCR amplification was carried out in a CFX Connect Real-time PCR system (Bio-Rad, U.S.A.) by using the SYBR Green Real-time PCR Master Mix (Bio-Rad, U.S.A.) in 20 μl reaction mixture. Primers used in qRT-PCR are listed in Table S2. The 2^(-ΔΔCT)^ method was used to analyze the relative quantification of gene expression (Livak and Schmittgen 2001). The data were expressed as the mean ± SD (three biological repeats, each with three technical repeats).

### RNA-seq analysis

Total RNA samples were extracted from the younger and older leaves of ZS9 and *yvl*. The time of sample collection for RNA-seq was in accordance with the determination of chlorophyll content, chloroplast ultra-structure. The transcriptomes were sequenced on an Illumina HiSeq2500 platform (Illumina, U.S.A.). Comparing 218.8 million clean reads of the samples with the *B. napus* reference (http://www.genoscope.cns.fr/brassicanapus/data/), total 147.7 million reliable mapped reads were obtained for RNA-seq analysis (Figure S1). The cluster method was used to calculate the distance in biological repeats. Levels of gene expression were calculated using the fragments per kilo base transcript per million reads (FPKM) method (Trapnell *et al.* 2010). The significance level of differentially expressed genes (DGEs) was determined using *p*-values < 0.05 and |log_2_ (Fold Change) | > 1.

### Statistical analysis

All statistical analyses in this study were performed using Student’s *t*-test. Each diagram was annotated with the number of biological repeats in each experiment. Values were considered as significantly different with the threshold of *P < 0.05 and **P < 0.01.

### Data availability

Figure S1 describes RNA-seq analysis of ZS9 and *yvl*. Figure S2 shows polymorphic molecular markers. Figure S3, S4 and S5 illustrate sequence alignment of three genes in which SNP variation occurred. Figure S6 and S7 illustrate amplification and digestion by *Bln* I of ORF10. Figure S8 illustrates the domain of CHLH protein in multiple species. Figure S9 shows FPKM value of *BnaA03.CHLH* in ZS11 tissues. Figure S10 illustrates the expression of chlorophyll synthesis genes. Table S1 lists the information of 629 rapeseed accessions. Table S2 lists all the primer sequence in this study. Table S3 describes the comparison of agronomic traits between ZS9 and *yvl* mutant. Table S4 describes chlorophyll contents of younger and older leaves between ZS9 and *yvl*. Table S5 shows the statistics of whole genome re-sequencing in BSA-seq study. Table S6 and S7 respectively list the functional annotation of candidate genes and genes transcribed by PEP and NEP. Supplemental material available at figshare: https://doi.org/10.25387/g3.12625796.

## Results

### Phenotypic characterization of yvl mutant

**Figure 1:**
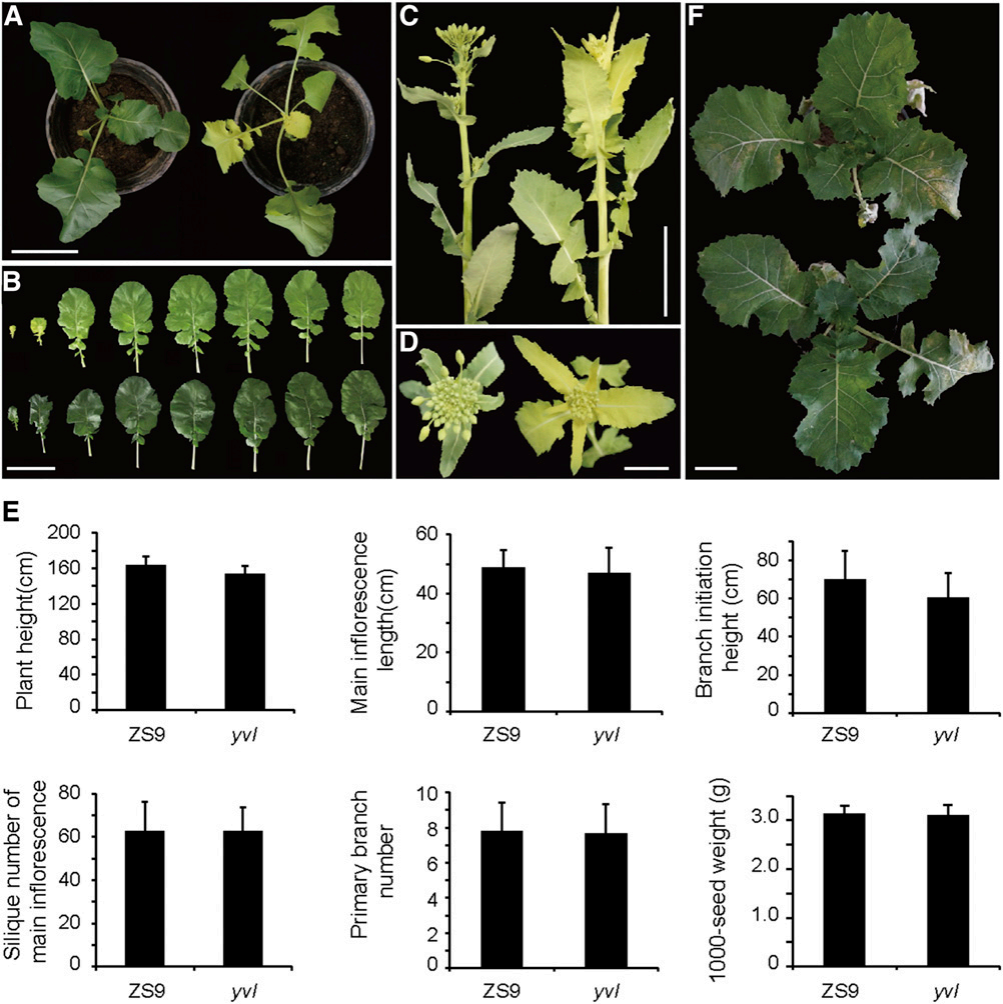
Phenotypic characterization of the *yvl* mutant. (A) Phenotypes of ZS9 (left) and *yvl* (right) at 5 weeks after sowing. Scale bar = 10 cm. (B) Leaf color of *yvl* (top) and ZS9 (bottom). Scale bar = 10 cm. (C) and (D) Leaf color of ZS9 (left) and *yvl* (right) at the bud stage. Scale bar = 5 cm (C) and 2 cm (D). (E) Phenotypes of the reciprocal F_1_ from crosses of *yvl* and ZS9 at 8 weeks after sowing. Scale bar = 10 cm. (F) Statistical analysis of agronomic traits between ZS9 and *yvl*. Thirty plants were measured. Error bars indicate SD.

The *yvl* mutant was obtained from EMS-induced *B. napus* ZS9. The younger leaves of mutant plant were clearly distinguished by a yellowish leaf color phenotype (Figure 1A, B). With plants growing, the younger leaves remained yellowish at the cotyledon stage and gradually became virescent toward maturity (Figure 1C, D). At maturity, the agronomically important traits showed only slight but non-significant differences between the mutant and wild type plants. Plant height and branch initiation height were slightly shorter in mutant plants but the differences were statistically insignificant (Figure 1F and Table S3).

### Physiological and anatomical features of yvl mutant

**Figure 2:**
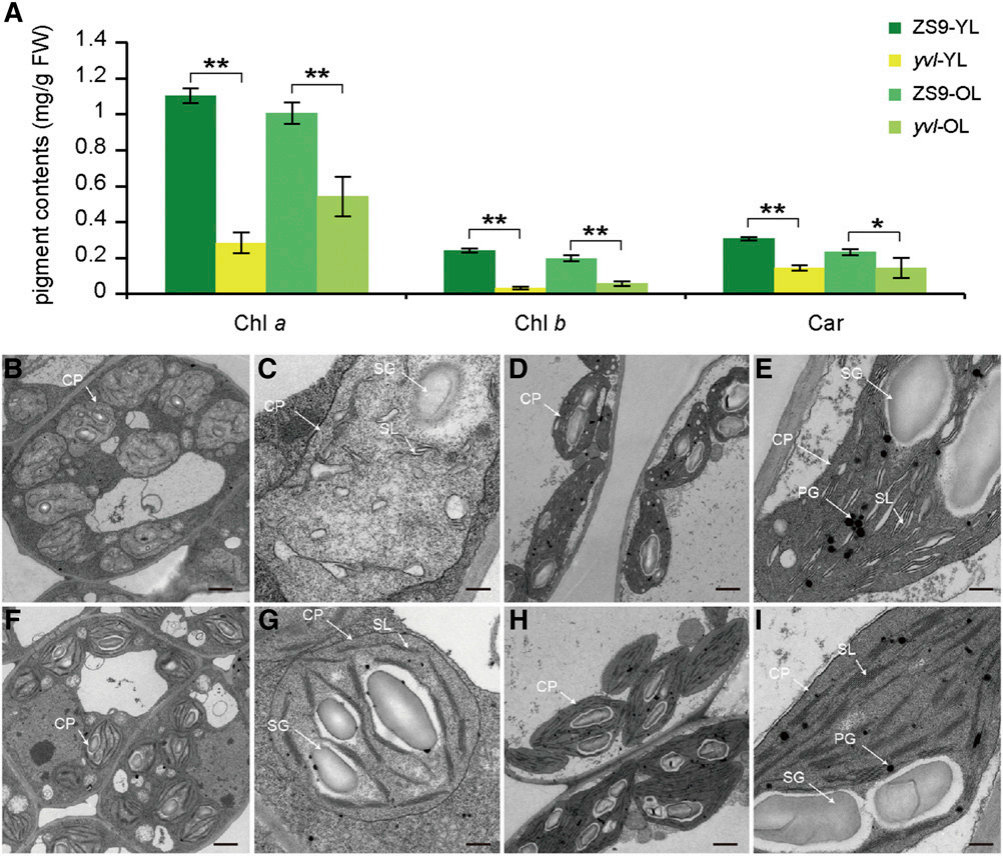
Analysis of chlorophyll contents and ultra-structure of chloroplasts. (A) chlorophyll content in younger leaves (YL) and older leaves (OL) of ZS9 and *yvl* mutant, in mg/g fresh weight. Error bars indicate SD from three independent repeats, significance level of Student’s *t*-test: *P < 0.05 and **P < 0.01. (B-I) Ultra-structure of chloroplasts in mesophyll cells of younger and older leaves from ZS9 and *yvl*. (B-C) younger leaf sample of *yvl*; (F-G) younger leaf sample of ZS9; (D-E) older leaf sample of *yvl*; (H-I) older leaf sample of ZS9. Each sample (younger and older leaf) in ZS9 and *yvl* consists of 12 photographs of three biological repeats. Scale bar = 500 nm (C, E, G, I) and 2 μm (B, D, F, H). CP: chloroplast, SG: starch grain, PG: plastoglobule, SL: stroma lamella.

Since the yellowish (chlorotic) leaves are associated with disruptions of chlorophyll biosynthesis, we estimated the chlorophyll content of younger and older leaves from the *yvl* mutant and ZS9 plants to determine the difference in pigment accumulation of the two. We observed that chlorophyll a (Chl *a*), chlorophyll b (Chl *b*) and carotenoid (Car) levels were remarkably lower in both younger and older leaves of *yvl* than in ZS9 (Figure 2A and Table S4). In the older leaves, the pigment levels seemed to recover partially but were still significantly lower in than in the wild type (Figure 2A and Table S4). This would suggest a disruption of chlorophyll biosynthesis pathway throughout the plant development.

To further investigate the effects of mutation of the chloroplast, we observed the ultrastructure of the chloroplast in both mutant and wild type under Transmission electron microscopy (TEM). The chloroplasts in ZS9 contained well-developed lamellar structures equipped with normally stacked grana and thylakoids regardless of younger and older leaves (Figure 2F-I). However, the structure of chloroplasts in *yvl* younger leaves was anomalously shaped with several vesicles instead of thylakoids (Figure 2B, C). The density and shape of chloroplasts of older leaves in *yvl* were almost similar with ZS9 leaves, but again, the granum was accumulated with abnormally developed lamellar structures composed of thylakoid membranes and lacuna (Figure 2D, E). The results suggested that the mutation (s) had clearly disturbed the chloroplast development and chlorophyll biosynthesis, which can ultimately affect the photosynthetic capacity of the plants.

### Rapid delimitation of a candidate genomic region by BSA-seq

For genetic analysis of the *yvl* locus, two crosses ZS9 × *yvl* and *yvl* × ZS9 were developed. The reciprocal F_1_ plants from the two crosses exhibited ZS9-like phenotypes (Figure 1E). The F_2_ segregation pattern fitted a 3:1 ratio of ZS9- to *yvl*-type plants (χ^2^ < χ^2^_0.05_ = 3:84; *P* > 0.05) (Table 1). In addition, the BC_1_ progenies showed an expected Mendelian inheritance ratio 1:1 (χ^2^ < χ^2^_0.05_ = 3:84; *P* > 0.05) (Table 1). These data indicated that the phenotype of *yvl* mutant was controlled by a single recessive nuclear gene.

**Table 1:** Segregation of F_2_ and BC_1_F_1_ populations

Cross	ZS9×*yvl*	*yvl*×ZS9	(*yvl*×ZS9) ×*yvl*
Number of ZS9-type plants*^a^*	719	261	75
Number of *yvl*-type plants*^b^*	208	85	84
Total plants	927	346	159
ZS9-type/*yvl*-type	3.45	3.07	0.89
Excepted	3:1	3:1	1:1
χ^2^*^c^*	3.11	0.02	0.40

a,b ZS9-type plants and *yvl*-type plants were determined by visual inspection.

c χ^2^ < χ^2^_0.05_ = 3.84, *P* > 0.05 was considered statistically significant.

As for the BSA-seq, 268.83 million paired-end reads of two parental lines (133.73 million reads for ZS9, and 135.10 million reads for *yvl*) were generated with 97.29% and 98.43% mapping rate, 19.84 × and 23.49 × sequencing depth and 91.43% and 92.73% genome coverage for ZS9 and *yvl*, respectively (Table S5). Similarly, the G- and Y-pools had an alignment of 30.43 × and 26.96 × sequencing depth, and 95.99% and 94.34% genome coverage, respectively (Table S5). The statistics results suggested that the WGS data were reliable and could be used for subsequent mutation detection and BSA-seq analysis.

**Figure 3:**
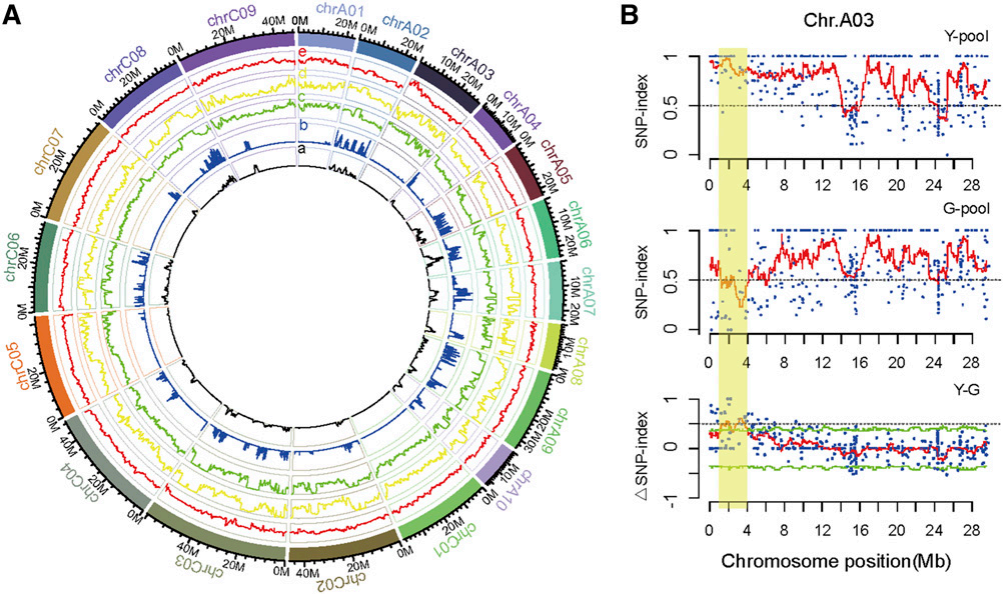
BSA-seq approach applied for mapping genomic region. (A) The innermost two circles (a and b) respectively denoted genome-wide densities of SNPs and InDels between ZS9 and *yvl* based on WGS data. The outermost circles denoted the different physical size of 19 chromosomes. The green (c) and yellow (d) circles displayed the SNP-index of *yvl*-type leaf color pool and ZS9-type leaf color pool respectively. The red (e) circle showed ΔSNP-index. (B) SNP index plot of *yvl*-type leaf color pool (top), ZS9-type leaf color pool (middle) and ΔSNP-index plot (bottom) of chromosome A03. The significant genomic region (1.0–4.0 Mb) was highlighted in shaded yellow color.

Based on the results of genotyping and filtration, the homozygous 182,078 SNPs and 91,553 InDels polymorphic markers were obtained from the two parental lines. The density of SNPs and InDels in *yvl* revealed that the variations were evenly distributed throughout the whole genome (Figure 3A). The visualized SNP-index plots of G- and Y-pools were delineated, followed by the depiction of ΔSNP-index. The SNP-index plots were similar across the entire genome for the two pools, except for a single region on chromosome A03 ranging 1.0∼4.0 Mb (Figure 3A, B). Therefore, this region on chromosome A03 was considered as a unique candidate region of the *yvl* locus (Figure 3B).

### Map-based cloning of the yvl gene

**Figure 4:**
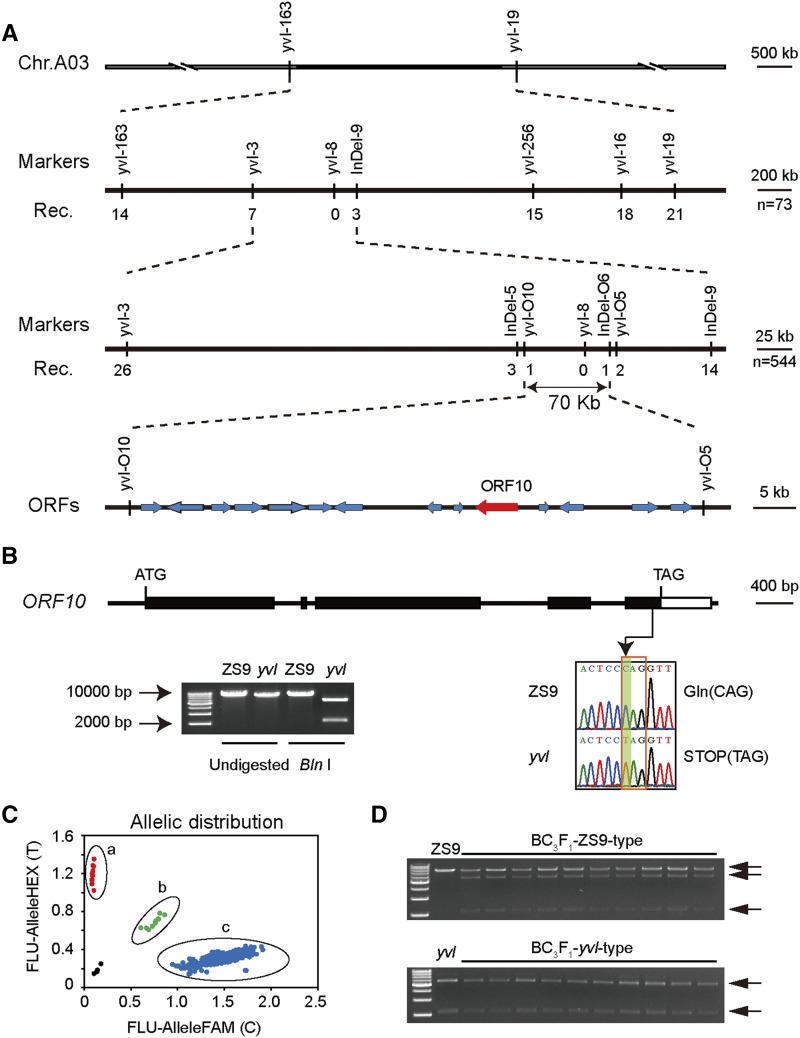
Map-based cloning of the *yvl* gene. (A) The *yvl* locus was initially mapped to a 3.0 Mb of chromosome A03 based on BSA-seq. Finally the *yvl* locus was limited to a 70-kb region linked with yvl-O10 and InDel-O6 using 73 F_2_ and 544 BC_2_F_2_ mutant phenotype individuals. (B) Gene structure of *BnaA03.yvl* and confirmtion the mutantion site by Sanger sequencing and enzyme digestion analysis. The positions of ATG and TAG are the start and stop codons. Black boxes and white box represent exons and 3′ UTR region, respectively. Green shade and red box indicates the ‘C’ to ‘T’ substitution and codon change. Verify the change with CASP marker by *Bln* I digestion. The original full-length gel was displayed in Figure S6. (C) SNP variation among 629 rapeseed accessions and 20 BC_3_F_1_ plants. Red, green, and blue dots represent three types SNP variation (T:T, T:C, and C:C). Black dots are the negative control. (a) *yvl* mutant and 10 *yvl*-type BC_3_F_1_ plants; (b) 10 ZS9-type BC_3_F_1_ plants; (c) 628 rapeseed accessions. (D) 10 *yvl*-type (the bottom) and 10 ZS9-type (the top) of BC_3_F_1_ plants digested by *Bln* I. *yvl* and ZS9 are the control in the two gels respectively. The grouping of gels were cropped from different parts of the same gel and the original full-length gel was displayed in Figure S7. The marker used in (B) and (D) was 1kb ladder marker.

According to the data of WGS, eleven positive molecular markers, including 5 SNP and 4 InDel markers in the candidate region, as well as 2 SNP flanking markers, were developed (Figure S2). Utilizing the developed markers and 72 F_2_ homozygous *yvl*-type individuals, the *yvl* locus was narrowed down to 591-kb on chromosome A03 between marker yvl-3 and InDel-9 (Figure 4A). Further mapping using 544 BC_2_F_2_ *yvl*-type individuals and linked markers indicated that the *yvl* locus was restricted to a 70-kb region flanked by yvl-O10 and InDel-O6 (Figure 4A).

Based on the annotations of *B. napus* genome database (http://www.genoscope.cns.fr/brassicanapus/data/), this region comprised of 14 putative genes with annotated functions (Figure 4A and Table S6). The alignment showed no sequence variations in the coding region between ZS9 and *yvl* of other *ORFs* except *ORF5*, *ORF10* and *ORF14* (Figure S3-S5). The SNP variations that occurred in *ORF5* and *ORF14* were synonymous mutations. Whereas, the mutation at SNP marker yvl-8, which co-segregated with the *yvl* phenotype, was a T substitution C in *BnaA03g04440D*, generating a premature stop codon and a Cleaved Amplified Polymorphism Sequences (CAPS) marker of the restriction enzyme *Bln* I, resulting in excluding 11 amino acids and the entire 3′ UTR region (Figure 4B). According to the function annotation of the *B. napus* genome database, *BnaA03g04440D* contains five exons, encoding a 1381 amino acid residue polypeptide, which is a putative large subunit H of Mg-protoporphyrin IX chelatase (CHLH), involved in chlorophyll biosynthesis and plastid-to-nucleus signal transduction.

We genotyped 10 homozygous *yvl*-type and 10 heterozygous ZS9-type individuals derived from BC_3_F_1_ were genotyped by this causal SNP substantiating the allelic variation (Figure 4C, D). The causal SNP polymorphism within *ORF10* was found to be perfectly consistent with the normal leaf color among the 628 accessions including ZS9 (Figure 4C and Table S1), suggesting that this site maybe highly conservative in *B. napus*. Taken together, these results suggest that *BnaA03.CHLH* is the most likely candidate gene responsible for the *yvl* phenotype.

### Characterization of BnaA03.CHLH

**Figure 5:**
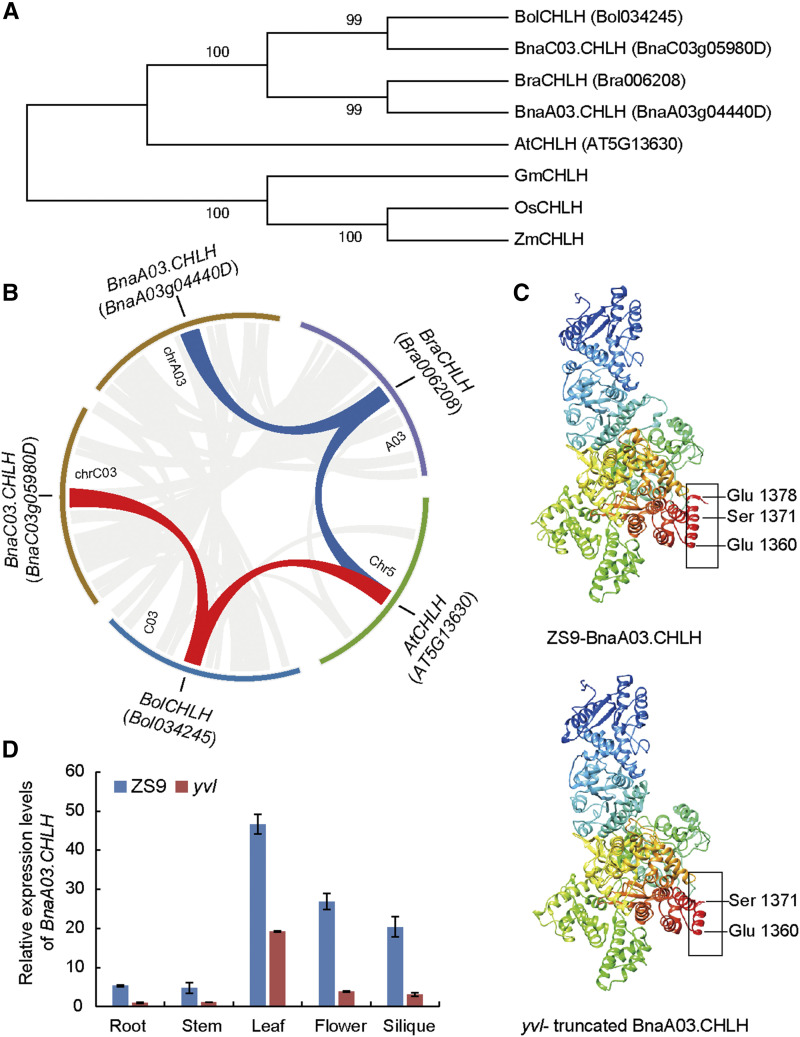
Characterization of *BnaA03.CHLH*. (A) Phylogenetic analysis of CHLH homologs in *Brassicaceae* species and *Oryza sativa*, *Zea mays*, *Glycine max*. (B) Synteny analysis of *CHLH* in *Arabidopsis*, *B. rapa*, *B. oleracea* and *B. napus*. (C) Protein conformation prediction of BnaA03.CHLH in ZS9 and *yvl*. (D) Expression pattern of *BnaA03.CHLH* in root, stem, leaf, flower, and silique of ZS9 and *yvl* based on qRT-PCR.

To detect the homologous genes of *BnaA03.CHLH* in *B. napu*s and their evolutionary relationship, a phylogenetic tree was constructed including *Brassicaceae* species and four other representative species. Phylogenetic analysis clearly distinguished monocotyledons (*Oryza sativa* and *Zea mays*) from dicotyledons (*Glycine max* and *Brassicaceae*) (Figure 5A). Bootstrap values indicate that *CHLH* is a conserved gene, widespread in monocotyledons and dicotyledons (Figure 5A). In *B. napus*, *BnaC03.CHLH*, as a homologous gene of *BnaA03.CHLH*, is present on chromosome C03. Moreover, *BnaA03.CHLH* and *BraCHLH* of *B. rapa* are orthologous genes and have a similar relationship as *BnaC03.CHLH* and *BolCHLH of B.oleracea*. In *Brassicaceae* species, the evolutionary relationship of *CHLH* conforms with U’s triangle (Figure 5A). At the chromosomal level, the syntenic regions harboring *CHLH* genes in *Arabidopsis*, *B. rapa*, *B. oleracea* and *B. napus* were delineated and the result of syntenic relationship was in accordance with the evolutionary relationship (Figure 5B).

It was reported that CHLH consists of two functional regions, the N-terminal region and cage-like assembly region, which are respectively composed of domains I-II and domains III-VI, as well as domain VI is a tight α-helix bundle in C-terminus (Chen *et al.* 2015). The sequence alignment of CHLH in monocotyledons (*Oryza sativa*, *Zea mays*) and dicotyledons (*Glycine max*, *Arabidopsis thaliana*, *B. rapa*, *B. oleracea and B. napus*) showed that the mutant SNP in domain VI was conserved (Figure S8). And then, we analyzed the protein structure prediction of CHLH in an online software Phyre^2^ (http://www.sbg.bio.ic.ac.uk/phyre2/html/). The conformation of CHLH protein in ZS9 showed that the last α-helix of C-terminus was from 1360 (Glu) to 1378 (Glu) in the amino acid sequence; however, all amino acids were deleted after 1371 (Ser) in *yvl*, resulting in a deformed/truncated CHLH protein in *yvl* (Figure 5C).

To investigate the expression pattern of *BnaA03.CHLH* in detail, its transcription levels in the root, stem, leaf, flower and silique tissues of ZS9 and *yvl* were analyzed by qRT-PCR. The expression level of *BnaA03.CHLH* was highest in the leaf, followed by flower, silique, root and stem in a descending order, which could be understandable due to an abundance of chloroplasts in the green plant tissues either in ZS9 or *yvl* (Figure 5D). In addition, it expressed higher in all tissues of ZS9 than in *yvl* (Figure 5D). Apart from qRT-PCR, we further analyze the expression pattern of *BnaA03.CHLH* using RNA-seq data of Zhongshuang11 tissues (including root, stem, leaf, bud, silique, sepal, ovule, pericarp and callus), previously published by our group (Li *et al.* 2019; Yao *et al.* 2020). The transcript abundance level of *BnaA03.CHLH* in leaf was consistent with the relative expression patterns observed in qRT-PCR (Figure S9). Thus, the expression pattern of *BnaA03.CHLH* showed consistency with the leaf color changes, further validating the candidacy of this gene for mutant phenotype.

### Mutation in BnaA03.CHLH degraded chloroplast structure and chlorophyll biosynthesis by limiting PEP activity

**Figure 6:**
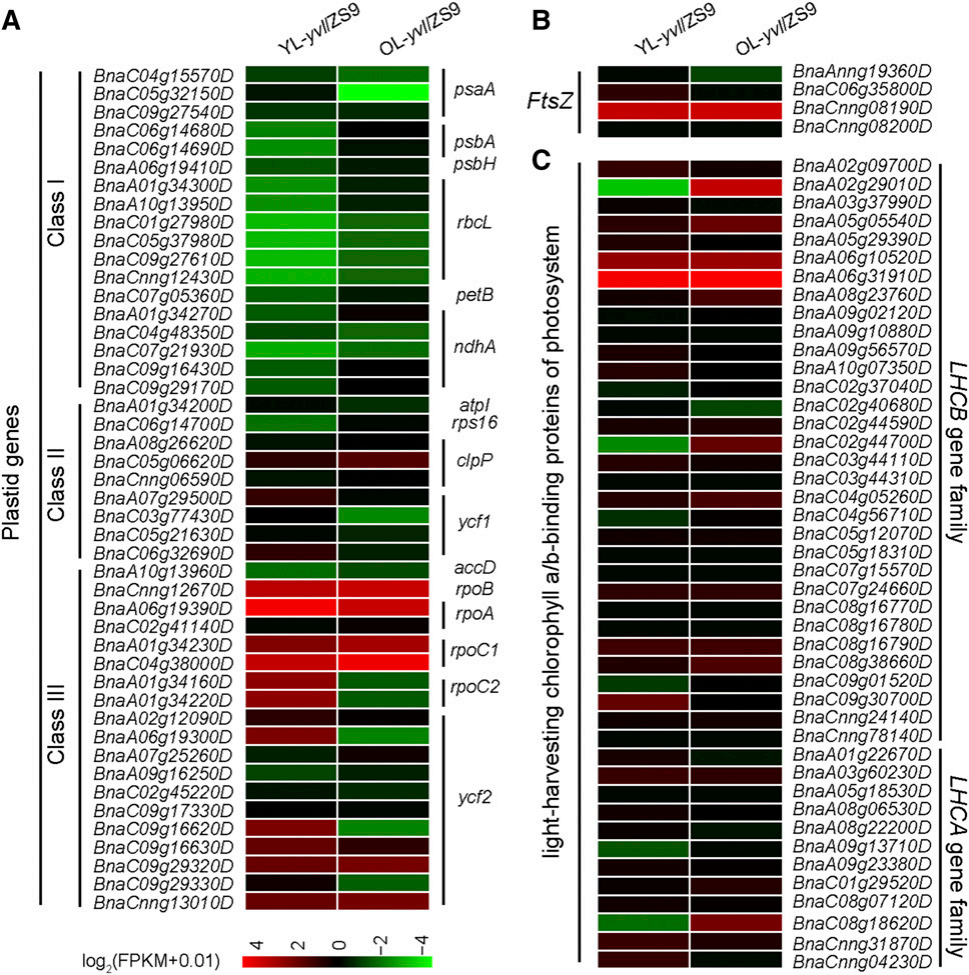
Transcriptional changes of differential expression genes between ZS9 and *yvl* based on RNA-seq data. (A) Heat map displaying the plastid-encoded genes expression levels of Class I: PEP transcribes genes, Class II: Plastid genes are transcribed together by both PEP and NEPs, and Class III: NEP transcribes genes. (B) Heat map displaying the homologous genes of *FtsZ*. (C) Heat map displaying the expression trend of *LHCA* and *LHCB* gene families by RNA-seq. The gene expression in the heat maps are Log_2_ [fold (RPKM+0.01)].

**Figure 7:**
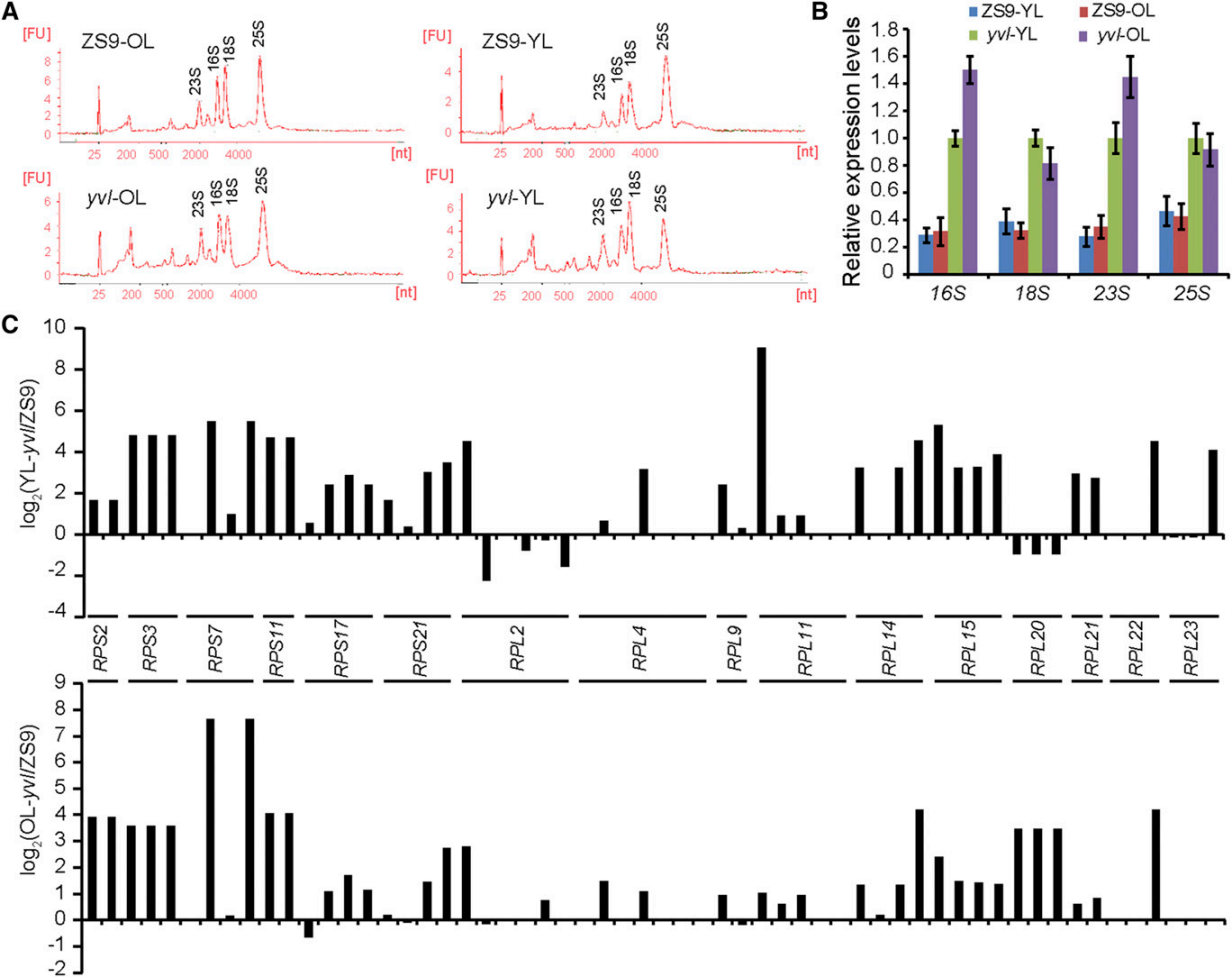
The rRNA analysis and expression levels of chloroplast ribosomal protein gene. (A) rRNAs analysis by Agilent 2100. (B) The expression levels of rRNAs in younger and older leaves of ZS9 and *yvl*. (C) Different expression of 58 chloroplast ribosomal protein genes according to RNA-seq data. The graph shows the log_2_ ratio of transcript levels in *yvl* compared with ZS9.

Chlorophyll content and chloroplast development were critically affected in *yvl* mutant. From RNA-seq data, we observed that the expression of class I plastid genes, which are transcribed by PEP was considerably reduced in the mutant plants, especially at the initial stages of development (Figure 6A and Table S7). The regulatory expression of other plastid and chlorophyll synthesis related genes was changed either slightly or insignificantly (Figure 6B and Figure S10). This indicated that the PEP activity may have been impaired to a greater extent, which led to the impairment of chloroplast development and chlorophyll degradation in *yvl*. Similar with these results, the decreased expression of class I plastid genes and increased expression of class III plastid genes was previously reported in *WP1* mutant, where the regulation of rRNA had also reduced significantly, especially in younger leaves (Wang *et al.* 2016c). Therefore, we also tested the rRNA levels in both younger and older leaves. rRNA analysis showed that there was no significant difference in the contents of 16S, 18S, 23S and 25S rRNA (Figure 7A). The expression levels of rRNA and ribosomal protein coding genes were slightly higher in *yvl* (Figure 7B, C). Therefore, we concluded that the chloroplast ribosome was not affected in the mutant, and the degradation of chloroplast ultrastructure occurred due to reduced PEP activity.

## Discussion

Yellow leaf mutants are often associated with disruptions of photosynthetic pathway, including the regulatory network of chlorophyll biosynthesis and chloroplast development genes. In the present study, we isolated a yellow-virescent leaf mutant in rapeseed and identified the causal mutant allele on chromosome A03 (*BnaA03.CHLH*). *CHLH* is a multifunctional gene involved in chlorophyll biosynthesis and the retrograde signaling pathway in higher plants (Adhikari *et al.* 2011; Chen *et al.* 2017). A previous study of *CHLH* mutant allele in *Arabidopsis* reported that mutations in the central protein domains (I to VI) resulted in reduction of Mg-chelatase activity and plastid signaling (Ibata *et al.* 2016). Here, we observed that the mutation in *BnaA03.CHLH* domain VI led to yellowish leaf color phenotype in young leaves and the mutation site was conservative in 629 rapeseed accessions (Figure 4B, C). The protein 3D structure showed that the last α-helix of the C-terminal region was incomplete in *yvl* mutant (Figure 5C), resulting in a yellowish phenotype. Mutants with yellowish leaf phenotypes are generally known to have degraded chlorophyll synthesis, which halts photosynthetic machinery of cells, ultimately leading to yield losses or cell death in severe cases. We observed reductions of chlorophyll content and chloroplast development predominantly in the younger leaves (Figure 2). In the older leaves of *yvl* mutant, the regulation of genes associated with chlorophyll synthesis, chloroplast development and light harvest chlorophyll a/b-binding (LHCA and LHCB) was not significantly inhibited (Figure 6A, C and Figure S10). This resulted in a delayed greenness in mutants, but the chlorophyll a/b and carotenoid contents were still considerably lower as compared to ZS9.

Delayed greenness has also been observed previously in other crops, for example, in rice, many leaf color mutants with yellowish younger leaves and greenish older leaves are reported (Wang *et al.* 2016a; Wan *et al.* 2015; Gong *et al.* 2014). The genes controlling leaf color in most of these mutants were identified to be essential for early chloroplast development, which is reasonable to explain the change of leaf color from yellow to green (Wu *et al.* 2007). Similarly, in this study, one assumption could be that the mutations in *BnaA03.CHLH* did not completely halt the chlorophyll synthesis process but slowed it down, and as the plants grew older, the chlorophyll synthesis recovered and a greenish leaf color phenotype was restored. Another plausible and rather interesting reason for the re-emergence of phenotype in older leaves could be associated to the gene expression patterns in polyploid genomes. In polyploid crops such as rapeseed, homologous expression bias and effects of dosage balance on gene expression are common phenomena (Yoo *et al.* 2014; Wang *et al.* 2006; Rapp *et al.* 2009; Stupar *et al.* 2007; Wu *et al.* 2018). For *BnaA03.CHLH* gene, we found a homologous gene on C-subgenome (Figure 5A, B). It could be assumed that redundant functional activity of the homologous gene *BnaC03.CHLH* restored the virescent leaf color phenotype in older leaves when the function of *BnaA03.CHLH* was halted due to the mutations in central protein domains. This could be studied further thoroughly in the future studies to understand the gene expression behavior of polyploid genomes.

Chlorophyll biosynthesis and chloroplast development are complex pathways and their regulatory mechanisms involve a multitude of genes and gene networks. Disruption in these pathways, even by point mutations, bring about changes in their entire regulatory networks, as has been reported in several studies in past (Wang *et al.* 2016c; Ge *et al.* 2017). Previous study of Wang and coworkers associated such phenotype changes in rice with an inhibitory expression of class-I plastid genes, which are transcribed by PEP. They further observed that the ribosomal proteins and rRNA levels were reduced in mutants, which resulted in degradation of chloroplast ultrastructure and chlorophyll biosynthesis (Wang *et al.* 2016c). We did not observe any considerable changes in the rRNA levels in chloroplast, but the class-I plastid genes including the regulatory genes of photosystem I and II were substantially reduced in the mutant plants. Since photosystem II is light harvesting complex and the initiating point of photosynthetic mechanism, reduced abundance of its regulatory transcripts caused disruptions in the entire pathway. Therefore, we conclude that a single nucleotide mutation in *BnaA03.CHLH* results in an imperfect PEP activity causing a reduced chlorophyll accumulation and a deformed chloroplast in rapeseed.
